# Molecular characteristics, phylodynamics, and evolutionary changes of avian infectious bronchitis virus detected from chickens in Yunnan Province, 2021–2024

**DOI:** 10.1016/j.psj.2026.107716

**Published:** 2026-09-03

**Authors:** Weiwu Mu, Huixin Liu, Xuzhao Zou, Ronghai Wang, Jingxia Wang, Xianghua Li, Fengping He, Sunpeth Angkititrakul, Bin Xiang, Liangyu Yang

**Affiliations:** a College of Veterinary Medicine, Yunnan Agricultural University, Kunming, 650201, China; b Center for Poultry Disease Control and Prevention, Yunnan Agricultural University, Kunming, 650201, China; c Faculty of Veterinary Medicine, Khon Kaen University, 40002, Thailand; d Yanjin County Bureau of Agriculture and Rural Affairs, Yunnan, Yanjin, 657500, China

**Keywords:** Avian infectious bronchitis virus, Recombination, Phylodynamics, Yunnan Province

## Abstract

Avian infectious bronchitis virus (IBV) is endemic in poultry flocks worldwide, posing a significant threat to the global poultry industry. Frequent mixing of free-range local chickens with introduced chickens in Yunnan Province, China, facilitates the transmission, recombination, and mutation of avian IBV, thereby complicating disease prevention and control. In this study, we aimed to investigate the presence of IBV in poultry populations in Yunnan Province. Samples were collected from live poultry markets (LPMs) and breeding farms, comprising 725 randomly sampled cloacal/fecal swabs and 55 tissue samples. IBV-positive samples were confirmed via polymerase chain reaction (PCR), with an overall positivity rate of 0.89% (7/780) for all tested samples. The positivity rate was 0.35% (2/564) in Kunming, 3.7% (2/54) in Zhaotong, 20% (1/5) in Yuxi, and 12.5% (2/16) in Baoshan, while no IBV was detected in samples from Lanping, Xichou, or Ninglang. Six IBV strains, including five GI-19 strains and one GVI-1 strain, were successfully isolated. Phylogenetic analysis further showed that the Yunnan GI-19 strains predominantly clustered with strains originating from Sichuan Province. Sequencing of the S1 gene revealed several amino acids substitutions per isolate in hypervariable regions HVR1–HVR3. Notably, a valine (V) and glycine (G) insertion between amino acid positions 88 and 89 was identified exclusively in isolate F210, a feature rarely reported in IBV. Protein-protein docking analysis indicated that the unique 88–89 insertion in isolate F210 S1 may alter its binding interactions with the host receptor ANPEP. Whole-genome comparison revealed that isolate YX3 shared 97.05% nucleotide identity with strain CK/CH/GX/YL17/2017 from Guangxi, whereas isolates Q47, F13, and F210 shared 96.40%–97.27% identity with strain CK/Henan/H1036/2021 from Henan. Recombination analysis detected obvious recombination events in isolates F13, F210, Q47, and YX3, with GI-22 strains serving as the major parental donors. These genetic characteristics, recombination patterns, and structural insights demonstrate the complex evolutionary dynamics of circulating IBV strains in Yunnan. Continuous molecular epidemiological surveillance combined with functional protein analysis is essential to monitor emerging variants and formulating targeted, effective disease control strategies.

## Introduction

Avian infectious bronchitis virus (IBV), a member of the genus *Gammacoronavirus* within the family *Coronaviridae*, is a highly contagious pathogen responsible for avian infectious bronchitis (IB). This viral disease is characterized by respiratory distress, reduced egg production, and renal damage in chickens (Xu et al., 2019). It poses a severe threat to the global poultry industry, causing significant annual economic losses due to its high transmissibility and rapid mutation rate (Liu et al., 2004; Zhou et al., 2017). The spike (S) protein of IBV, particularly the S1 subunit, serves as the primary antigenic component that mediates viral attachment and entry into host cells. The hypervariable regions (HVR1, HVR2, and HVR3) are tightly associated with antigenic variation, serotype diversity, and pathogenicity (Cavanagh et al., 1992; de Wit et al., 2011; Liu et al., 2009; Yan et al., 2021). These regions frequently undergo amino acid substitutions, insertions, and deletions, facilitating the emergence of novel IBV genotypes and serotypes capable of evading host immune responses, which further complicates IB prevention and control (Cook et al., 2012; Huang et al., 2020; Ji et al., 2020; Zhou et al., 2017). Newly emerged recombinant IBV strains can evade vaccine-induced immunity, potentially leading to vaccination failure in poultry flocks (Bali et al., 2021; Fellahi et al., 2015; Kim et al., 2023; Liu et al., 2014). Accordingly, novel IBV genotypes and serotypes continuously circulate and emerge globally. To date, phylogenetic classification has identified nine IBV genotypes (GI-1 to GIX-1) comprising 38 distinct genetic lineages (Christian Drosten et al., 2003; Jiang et al., 2017; Li et al., 2020; Ma et al., 2019; Mendoza-Gonzalez et al., 2022; Molenaar et al., 2020).

Due to its unique topography and diverse climate, Yunnan Province in China possesses abundant biological resources, supporting the formation of diverse indigenous chicken germplasm resources (Huo et al., 2014). Indigenous chickens in Yunnan are typically raised under free-range conditions and are often co-reared with introduced chickens—a practice that facilitates IBV spread and elevates the risk of recombination and genetic variation. Previous studies have reported that *coronaviruses* can transmit between poultry and wild birds, which potentially contributes to long-distance viral dissemination (Miłek et al., 2018). Wild birds are considered critical carriers responsible for the cross-regional spread of IBV variants, such as the QX variant (GI-19 lineage) from China to Europe and the Var2 variant (GI-23 lineage) from the Middle East to Poland (Miłek et al., 2018). Moreover, tens of thousands of migratory wild birds migrate southward to Yunnan for overwintering every autumn (Chen et al., 2024). Frequent ecological contact between these migratory birds and free-range indigenous chickens therefore creates favorable conditions for IBV cross-species transmission and regional dissemination (Fan et al., 2022). Thus, these complex ecological and breeding conditions make IBV prevention and control a persistent challenge for the poultry industry in Yunnan Province.

The GI-19 lineage has been identified as the predominant IBV genotype in several Chinese provinces (Huang et al., 2020; Xie et al., 2025), whereas GVI-1 and GI-22 strains are sporadically detected in domestic regions (Wang et al., 2024; Zhao et al., 2019). Recombinant IBV strains, such as I0718/17, I0722/17, I0724/17, and I0737/17, have also been reported (Xu et al., 2019). Nevertheless, their recombination patterns and evolutionary dynamics in Yunnan remain poorly understood. Moreover, unique amino acid insertions in IBV strains that may affect antigenicity and pathogenicity have rarely been documented in Yunnan, further underscoring the need for in-depth research.

Therefore, the present study was designed to isolate and identify IBV strains from chicken flocks in Yunnan, characterize the S1 gene, focusing on its hypervariable regions and amino acid variations, investigate recombination events and phylogenetic relationships among the isolates, and elucidate the epidemiological dynamics of IBV in this region. The findings will provide a critical knowledge gap regarding IBV in Yunnan, offer insights into its genetic evolution, and provide a scientific basis for developing targeted prevention and control strategies against IBV-associated diseases in the region.

## Materials and methods

### Sample collection and processing

From 2021 to 2024, a total of 725 cloacal/fecal swabs were randomly collected and 55 tissue samples were collected from poultry across Yunnan Province, China, during active epidemiological surveillance. All cloacal/fecal swab samples were immersed in 1.5 mL of phosphate-buffered saline (PBS) and stored at −80°C prior to laboratory processing.

### RNA extraction and cDNA synthesis

Total viral RNA was extracted using the RNA isolator Total RNA Extraction Reagent (R401-01, Vazyme, China) in accordance with the manufacturer’s standard protocols. First-strand cDNA was synthesized with random primers using the HiScript II 1st Strand cDNA Synthesis Kit (R211-01, Vazyme, China).

### PCR amplification and sequencing

The full-length S1 gene was amplified via PCR using a custom sense primer (5′-TTGAAAACTGAACAAAAGACCGT-3′) and an antisense primer (5′-TACAAAACCTGCCATAACTAACATA-3′). The 50 μL PCR reaction system consisted of 2 μL of template cDNA, 2 μL of each primer (10 pmol/L), 21 μL of nuclease-free water, and 25 μL of 2× Rapid Taq Master Mix (Vazyme, China). The PCR cycling protocol was set as follows: initial denaturation at 95°C for 3 min; 35 cycles of denaturation at 95°C for 15 s, annealing at 55°C for 15 s, and extension at 72°C for 15 s; followed by a final extension at 72°C for 5 min.

To acquire the nearly complete IBV genome sequence (excluding the 5′ and 3′ termini), multiple genomic fragments were amplified using IBV universal consensus primer sets (Supplementary Table S1), which were designed based on multiple sequence alignments of IBV genomes sequences. All genomic fragments were amplified under the same PCR conditions described above, with annealing temperatures individually optimized for each primer pair as listed in Supplementary Table S1. All PCR amplification products were visualized and verified via 1% agarose gel electrophoresis.

### Cloning and nucleotide sequencing

Target PCR products were purified using the Omega Gel Extraction Kit (Omega, Norcross, GA, USA). Purified DNA fragments were ligated into the pMD-19T cloning vector (Takara, Japan) and subsequently transformed into *E. coli* DH5α competent cells (AlpaLifeBio, Shenzhen, China). Positive recombinant clones were screened by colony PCR using the corresponding primer pairs and then valid clones were subjected to bidirectional Sanger sequencing by Sangon Biotech (Shanghai, China).

### Phylogenetic analysis

Raw sequence editing, quality trimming, and sequence assembly were performed using SeqMan (version 5.01; DNAStar, Madison, WI, USA). In this study, four full-length genome IBV genome sequences and two complete S1 gene sequences were successfully obtained. All newly generated sequences were deposited in the GenBank database, with detailed accession information listed in Table 1.

**Table 1:** Detailed information of the IBVs isolated in this study.

Isolates	Origin	Isolation date	Subgroup	Accession no.
A/Chicken/Yunnan/F13/2021	Baoshan	2021	GI-19	PZ200906
A/Chicken/Yunnan/YX3/2022	Yuxi	2022	GIV-1	PZ200909
A/Chicken/Yunnan/F210/2021	Baoshan	2021	GI-19	PZ200907
A/Chicken/Yunnan/Q47/2022	Fuyuan	2022	GI-19	PZ200908
A/Chicken/Yunnan/YJ1/2024	Yanjing	2024	GI-19	PZ200910
A/Chicken/Yunnan/YJ2/2024	Yanjing	2024	GI-19	PZ200911

For phylogenetic characterization, the obtained S1 gene sequences were aligned with publicly available reference IBV sequences using the MUSCLE algorithm. Neighbor-joining(NJ) phylogenetic trees based on complete genomes and individual viral genes were constructed using MEGA 11.0 (www.megasoftware.net), with branch bootstrap support evaluated by 1,000 replicate analyses.

To further assess the genetic diversity and phylogenetic topology of the S1 gene, a maximum-likelihood (ML) phylogenetic tree was constructed using IQ-TREE integrated in PhyloSuite v1.2.2 (Zhang et al., 2020), and branch reliability was assessed via 10,000 ultrafast bootstrap replicates.

### Recombination detection

Seventeen representative IBV reference genome sequences were selected as putative parental strains for recombination analysis to identify recombination breakpoints and recombination events. Potential recombination events were initially predicted using RDP 4.0 software with nine embedded analytical methods, including RDP, GENECONV, BootScan, MaxChi, Chimaera, SiScan, PhyLPro, LARD, and 3Seq (Martin et al., 2015). All predicted recombination events were further validated and visualized using SimPlot v3.5.1.

### Protein structure preparation, docking, and interaction analysis

The S1 amino acid sequences of isolate F210 and the classical LX4 reference strain were obtained by sequencing and from GenBank (AY189157), respectively. LX4 was used as the reference for amino acid position numbering. Three-dimensional structures of the S1 proteins and chicken ANPEP (UniProt: O57579) were predicted using AlphaFold 3 (version 1.0), and models with global predicted local distance difference test (pLDDT) scores >90 were retained. The structures were prepared in Schrödinger Suite 2024-1, including protonation at pH 7.4 and restrained minimization with the OPLS4 force field to a heavy-atom RMSD of 0.3 Å. Protein–protein docking was performed using PIPER 2.0, with ANPEP designated as the receptor and the S1 proteins as ligands. A total of 70,000 rotations were sampled, and the 30 top-ranked poses were retained for each complex. The highest-ranked pose was used to examine interface residues and noncovalent interactions in Schrödinger Suite 2024-1, and the resulting complexes were visualized using PyMOL 2.5.

## Results

### Prevalence of infectious bronchitis virus in Yunnan Province

Between 2021 and 2024, a total of 725 swab samples and 55 tissue samples were collected from chickens in Yunnan Province. RT-PCR identified seven IBV-positive samples, including five swabs and two tissue samples, yielding an overall positivity rate of 0.89% (7/780). The positivity rates varied by location: 3.7% (2/54) in Zhaotong, 0.35% (2/564) in Kunming, 20% (1/5) in Yuxi, and 12.5% (2/16) in Baoshan. No positive samples were detected in Lanping, Xichou, and Ninglang. Four IBV-positive samples were selected for complete genome sequencing (isolates F13, F210, Q47, and YX3; live poultry markets), and two additional positive samples were selected for S1 gene sequencing (isolates YJ1 and YJ2; local chicken farms). The GenBank accession numbers for these sequences are listed in Table 1.

### Sequence alignment and phylogenetic analyses

**Fig. 1:**
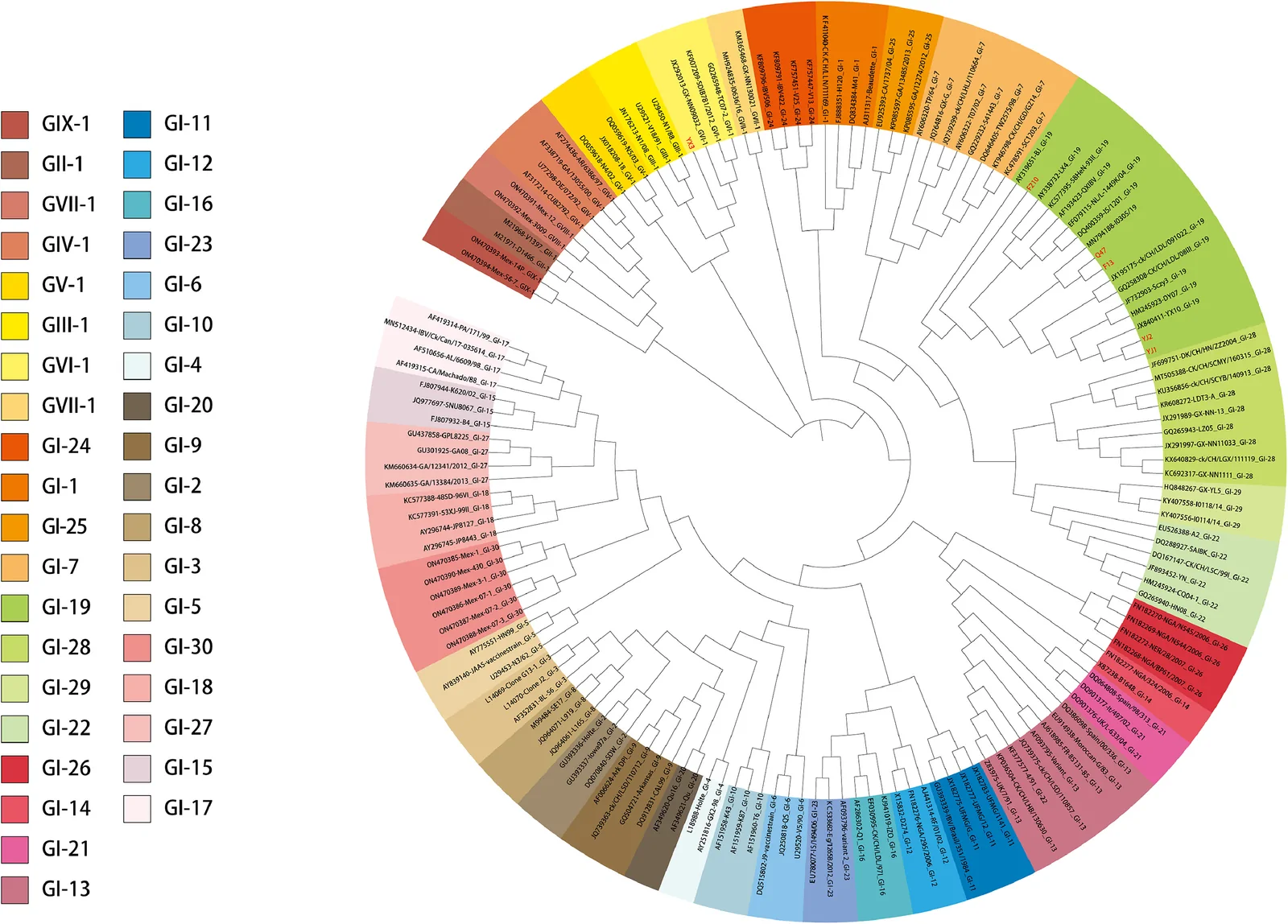
A phylogenetic tree of the S1 gene of the IBV strain isolated in this study and the reference strains has been constructed. This phylogenetic tree was constructed using the neighbor-joining method in MEGA11.0 software. The isolated IBV strain is marked with red.

Phylogenetic analysis based on the S1 gene (Fig. 1) classified five of the six Yunnan isolates as genotype GI-19 (F12, F210, Q47, YJ1, YJ2), and one isolate (YX3) as genotype GVI-1 (Table 1). Among the GI-19 isolates, pairwise nucleotide sequence identities ranged from 89.36% to 99.75%. Comparative analysis with reference sequences from GenBank revealed that isolates F13 and Q47 shared 99.38% nucleotide identity with strain CK/CH/HuB/X0329/2019 (Hubei, 2019). Isolates YJ1 and YJ2 showed 99.69%–99.75% identity with strain YX10 (Zhejiang, 2010). Isolate F210 exhibited 97.22% identity with strain CK/CH/JS/YC10.3 (Jiangsu), and isolate YX3 showed 99.38% identity with strain CK/CH/GD/JR12.4 (Guangdong).

### Genome sequence comparison

**Fig. 2:**
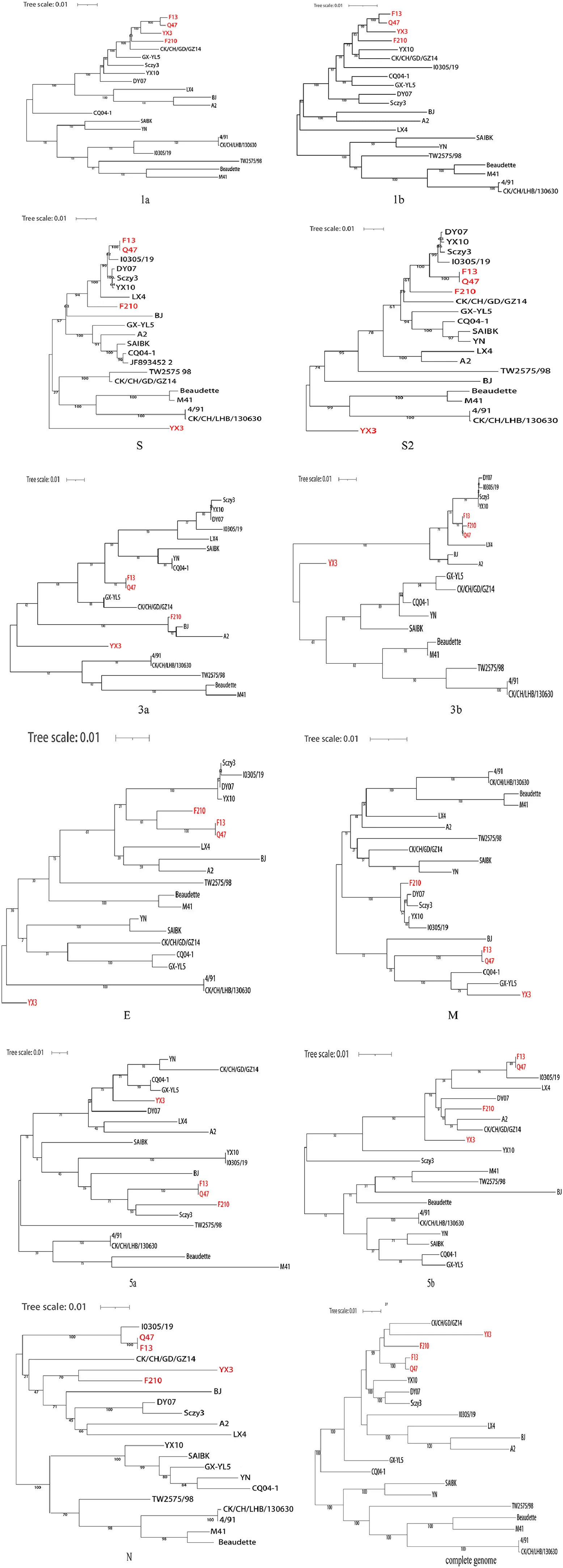
Phylogenetic trees based on the specific genes of the strains. These trees were constructed using the neighbor-joining method with bootstrapping for 1,000 replicates by MEGA11.0 software. The IBV isolation strains are marked with a red diamond.

Whole-genome comparison showed that isolate YX3 shared 97.05% nucleotide identity with strain CK/CH/GX/YL17/2017 (Guangxi), while isolates Q47, F13, and F210 shared 96.40%–97.27% identity with strain CK/Henan/H1036/2021 (Henan). However, analysis of individual genes revealed a mosaic pattern. For isolates Q47 and F13, the *1a, 1b, S, S2, 3a, 3b, 5a, 5b,* and *N* genes clustered with strains from Shandong (10305/19) and Henan (CK/Henan/H1036/2021), whereas their *E* and *M* genes were most closely related to the BJ strain. Isolate F210 showed a similar pattern, with most genes clustering with Shandong and Henan strains, but its *3a* and *E* genes were closest to BJ, and its *5b* gene was closest to the Guangdong strain CK/CH/GD/GZ14. Isolate YX3 had its *S, S2, 3a, 3b, E, M, 5a,* and *5b* genes clustered with the Guangxi strain CK/CH/GX/YL17/2017, while its *1a* and *1b* genes clustered with the Henan strain, and its *N* gene clustered with the Yunnan isolate F210 (Fig. 2).

### Genetic diversity of GI-19 S1 gene

**Fig. 3:**
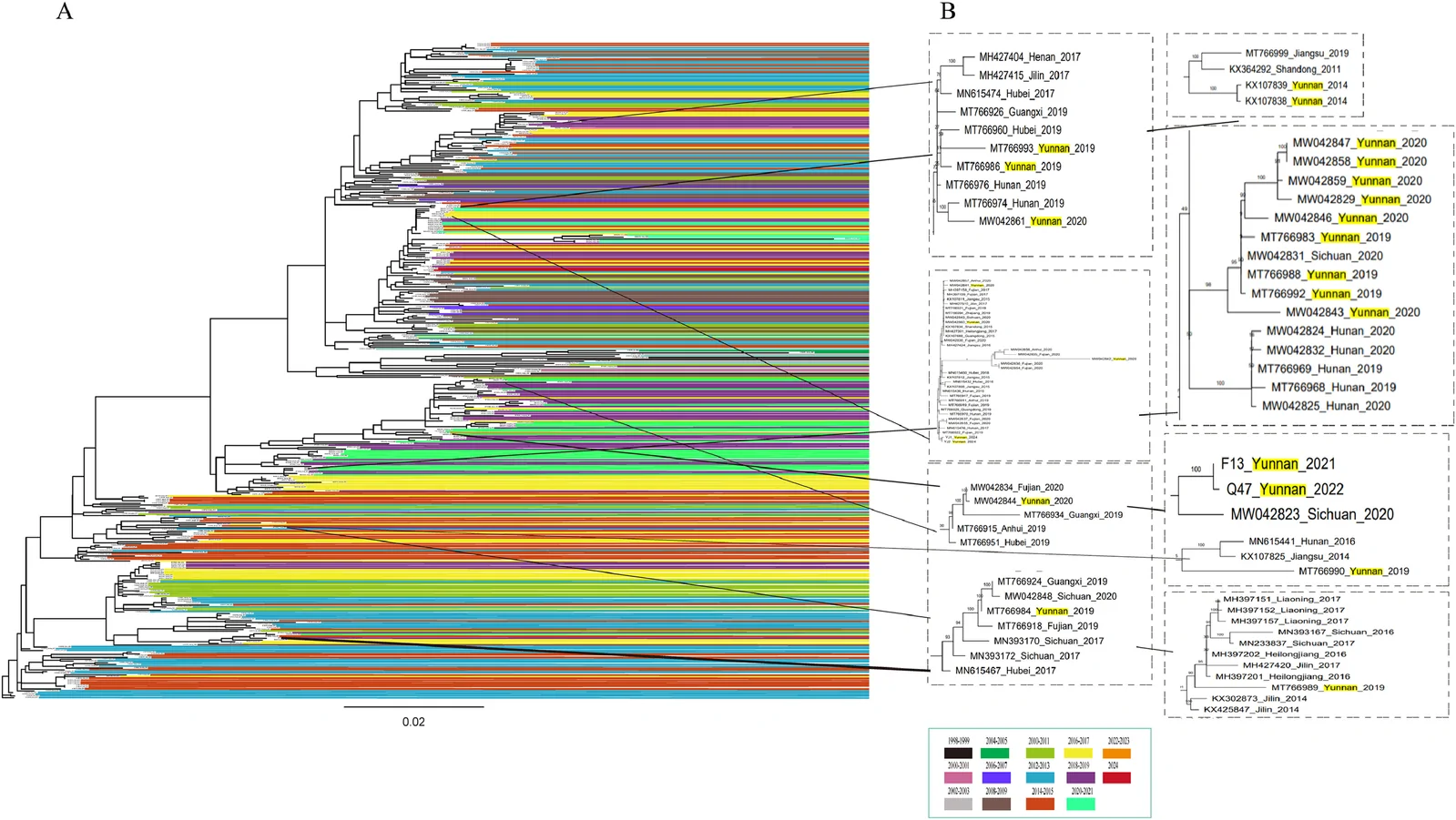
Phylogenetic tree of the genetic diversity of the S1 gene in the GI-19 type. Phylogenetic analysis was conducted using IQ-TREE integrated in PhyloSuite_v1.2.2. The maximum likelihood (ML) tree was inferred with 10,000 replicates of ultrafast bootstrap to assess branch support.

**Fig. 4:**
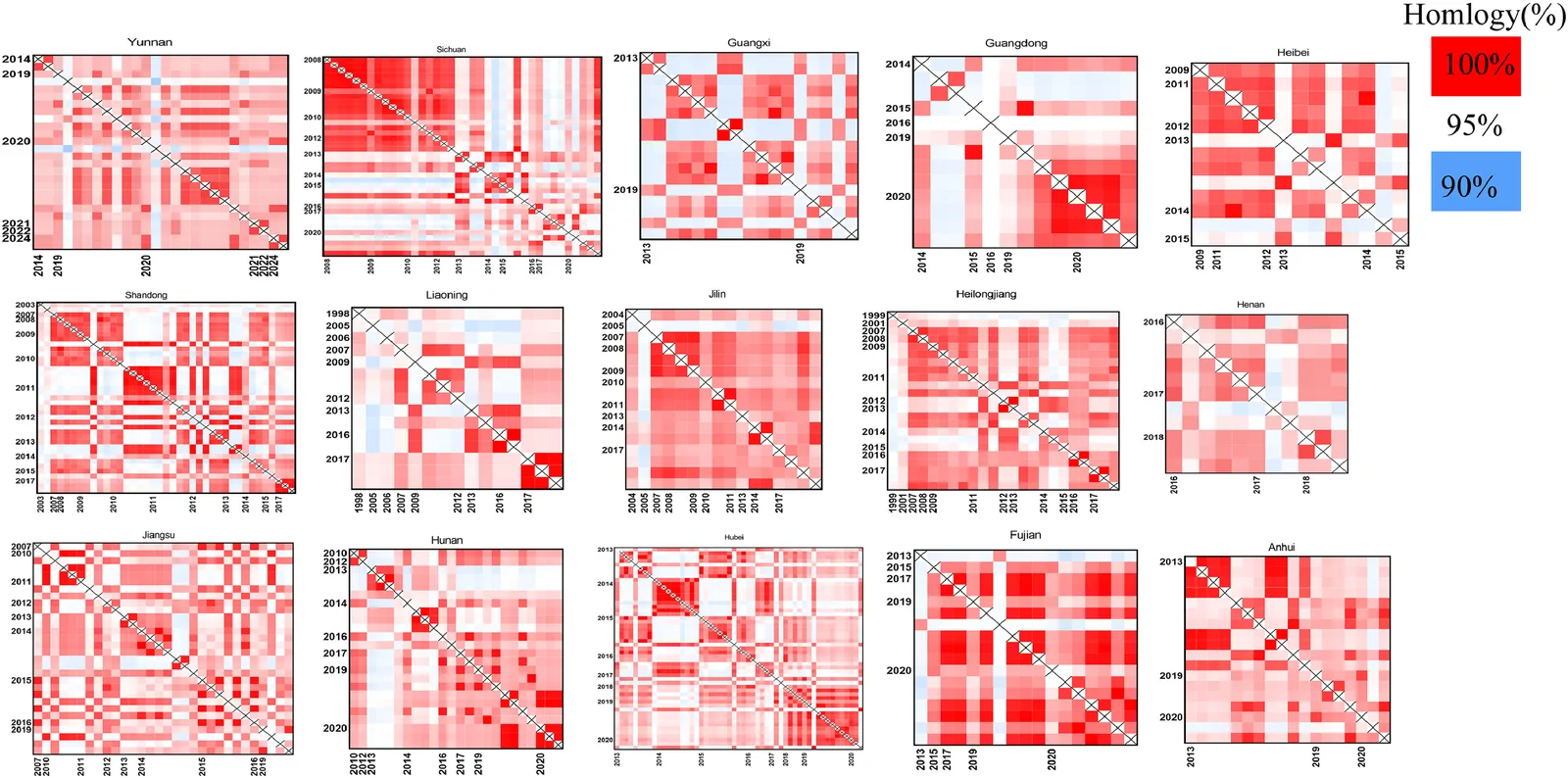
Homology of GI-19 isolates from various regions of China.

Phylogenetic analysis of the *S*1 gene of GI-19 strains from different Chinese provinces (Fig. 3) showed that Yunnan isolates most frequently clustered with strains from Sichuan, followed by strains from Guangxi, Fujian, Shandong, Hebei, Hunan, and northeastern China (Heilongjiang, Liaoning, Jilin). Pairwise nucleotide identity among Chinese GI-19 strains ranged from 93.33% to 96.91% when compared with the early reference strain KX252791 (Liaoning, 1998). As summarized in Table 2 and Fig. 4, strains from central China (Henan, Hubei, Hunan), eastern China (Jiangsu, Shandong), and southwestern China (Yunnan, Sichuan) exhibited high overall identity (up to 99.88% among Yunnan isolates), whereas strains from southern China (Guangdong, Guangxi) and northeastern China (Heilongjiang, Jilin, Liaoning) showed relatively lower identity, with the lowest pairwise identity (92.96%) observed among Fujian isolates.

**Table 2:** Homology among GI-19 IBV isolates from various regions of China.

region	Homlogy
Anhui	94.32%−99.75%
Fujian	92.96%−97.16%
Gaungdong	93.77%−97.35%
Guangxi	93.58%−97.96%
Hebei	94.33%−98.09%
Heilongjiang	93.89%−95.92%
Henan	94.33%−99.2%
Hubei	95%−98.52%
Hunan	94.07%−99.07%
Jiangsu	94.88%−99.38%
Jilin	94.51%−97.1%
Liaoning	94.8%−96.55%
Shandong	94.33%−96.12%
Sichuan	93.83%−99.75%
Yunnan	94.14%−99.88%

### Amino acid sequence characteristics of the S1 protein

Comparison of the deduced amino acid sequences of the five GI-19 isolates (F13, F210, YJ1, YJ2, Q47) revealed that isolate F210 contained a unique valine (V) and glycine (G) insertion between positions 88 and 89 of the S1 protein (Supplementary Table S2). The S1 proteins of the other GI-19 isolates were 540 amino acids in length, corresponding to 1620 nucleotides. The GVI-1 isolate YX3 had a longer S1 protein of 546 amino acids (1638 nucleotides).

The number and distribution of amino acid substitutions in the hypervariable regions (HVR1–HVR3) varied among isolates (Supplementary Tables S2–4). Isolate F210 carried 21 substitutions (7 in HVR1, 11 in HVR2, 3 in HVR3). Isolates YJ1 and YJ2 each had 16 substitutions (8 in HVR1, 6 in HVR2, 2 in HVR3). Isolate F13 had 14 substitutions (8 in HVR1, 6 in HVR2, 3 in HVR3). Isolate Q47 had 17 substitutions (9 in HVR1, 6 in HVR2, 2 in HVR3). The GVI-1 isolate YX3 had 13 substitutions (5 in HVR1, 3 in HVR2, 5 in HVR3).

### Recombination analysis

**Fig. 5:**
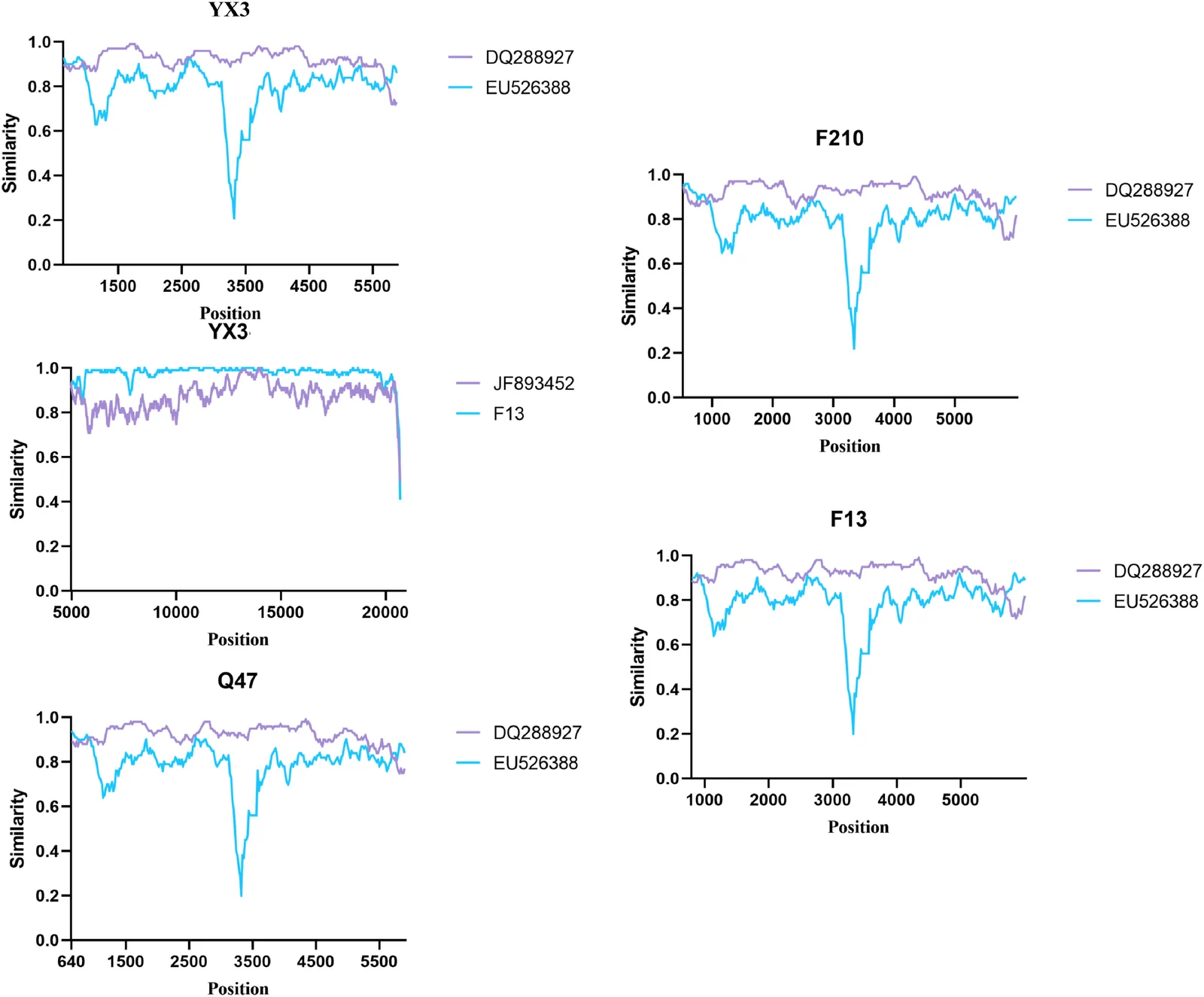
Recombination events in the gene from the isolated IBV strains.

Recombination analysis using RDP4 and SimPlot software identified several recombination events among the Yunnan isolates (Supplementary Table S5; Fig. 5). Isolate F13, a recombinant derived from A2-EU526388 (GI-22) as the major parent and SAIBK-DQ288927 (GI-22) as the minor parent, with recombination breakpoints at positions 941 and 5681. Isolate F210 showed the same parental combination with breakpoints at positions 901–5701. Isolate Q47 also exhibited a recombination event involving the same parental strains, with breakpoints at positions 941 and 5681. Isolate YX3 was identified as a recombinant from two events: first, between A2-EU526388 (GI-22) and SAIBK-DQ288927 (GI-22) (breakpoints 961–5681); second, between YN-JF893452 (GI-22) and isolate F13 (breakpoints 5001–19841). Overall, GI-22 lineage strains frequently participated as parental donors in these recombination events.

### Protein–protein docking reveals differential involvement of residues 88–89 in S1–ANPEP interaction

Protein–protein docking simulations were performed to investigate the interactions between the S1 proteins of different IBV strains and the chicken ANPEP receptor. Among the 70,000 sampled conformations generated by the PIPER docking algorithm, the top-ranked poses with the lowest energy scores were selected for structural and interaction analyses.

**Fig. 6:**
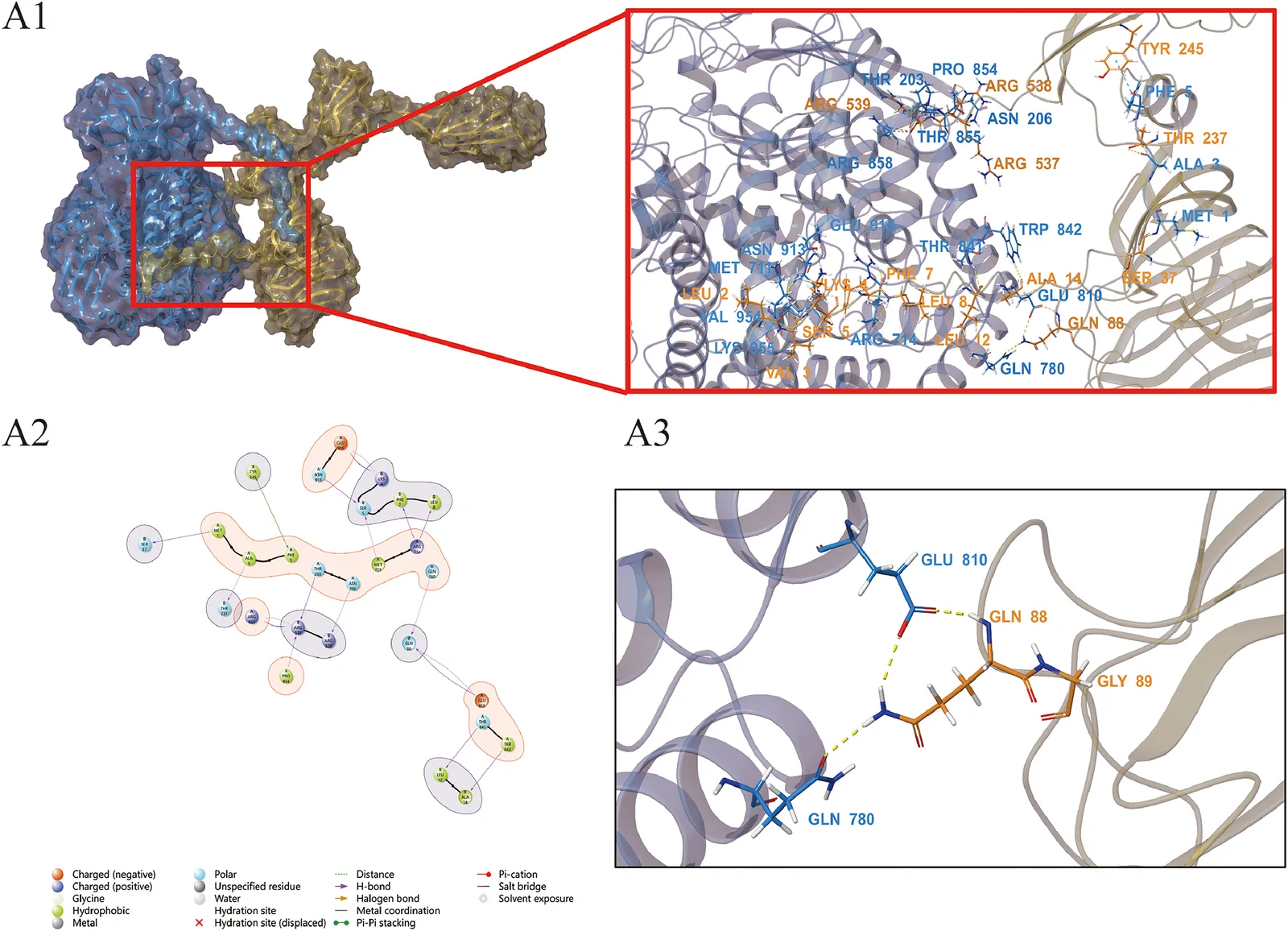
(A1) Three-dimensional representation of the complex between the host receptor ANPEP and the S1 protein of isolate F210.(A2) Two-dimensional interaction map of the docking pose (pose11) between ANPEP and the S1 protein of isolate F210.(A3) Detailed view of key amino acid interactions at the binding interface between ANPEP and the S1 protein of isolate F210.

For the ANPEP–F210 S1 complex, pose11 exhibited the lowest PIPER pose score (−450.931) and PIPER pose energy (−1134.122 kcal/mol), indicating the most favorable and stable binding configuration among all sampled conformations. Since PIPER energies below −1000 kcal/mol are generally considered indicative of highly stable protein–protein interactions, the ANPEP–F210 S1 interaction was regarded as highly stable. Three-dimensional structural visualization demonstrated a tightly associated complex between ANPEP (blue) and F210 S1 (yellow; Fig. 6). Detailed interaction analysis revealed that GLU915 of ANPEP formed both a hydrogen bond and a salt bridge with LYS4 of F210 S1, while GLU810 formed two hydrogen bonds with GLN88. In addition, a π–π interaction was identified between PHE5 of ANPEP and TYR245 of F210 S1, together with several hydrophobic and stabilizing interactions at the binding interface. Notably, residue 88 of the F210 S1 protein directly participated in receptor binding, suggesting a potential functional role for this region in ANPEP recognition.

**Fig. 7:**
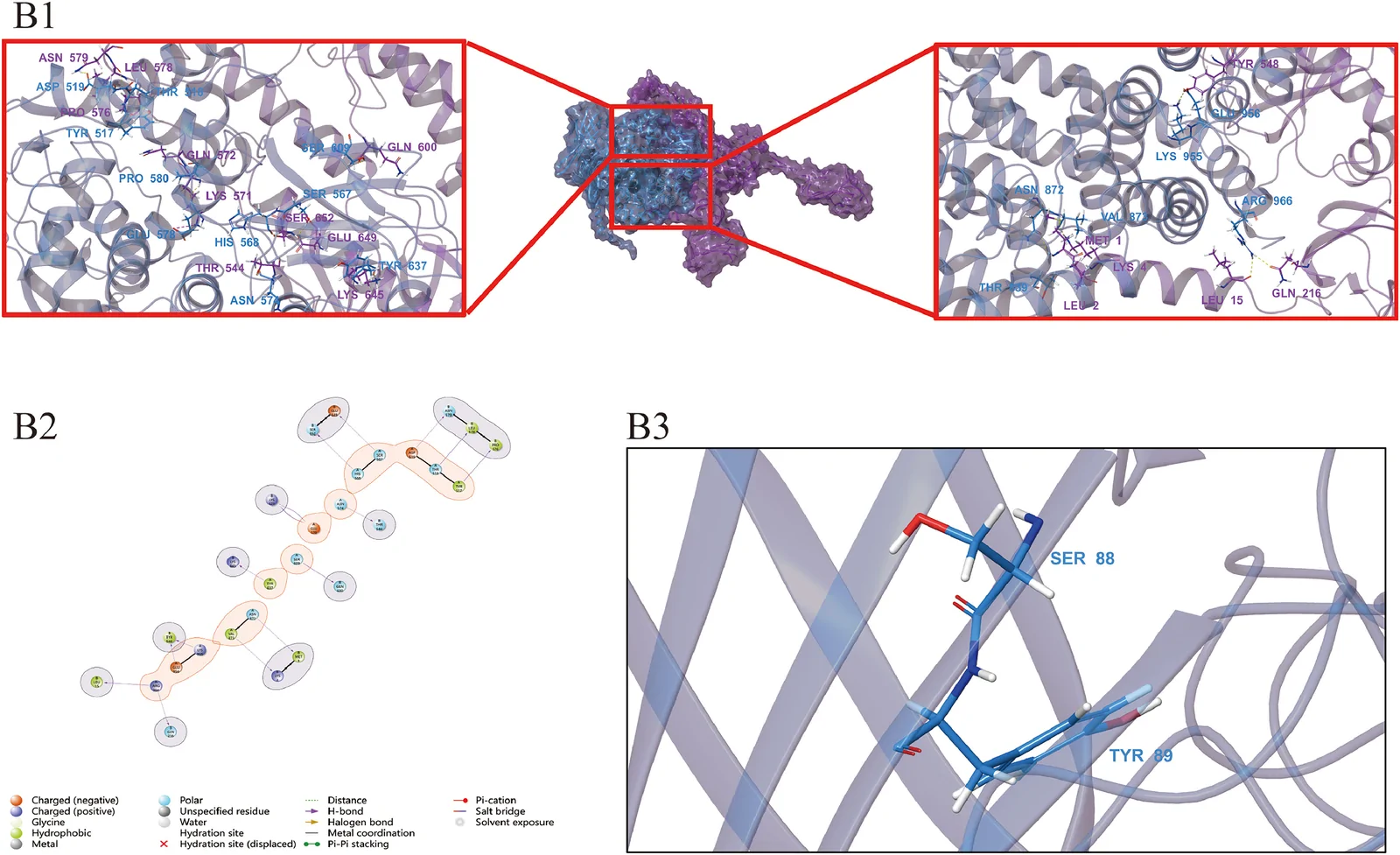
(B1) Three-dimensional representation of the complex between the host receptor ANPEP and the S1 protein of reference strain LX4. (B2) Two-dimensional interaction map of the docking pose (pose11) between ANPEP and the S1 protein of reference strain LX4. (B3) Detailed view of key amino acid interactions at the binding interface between ANPEP and the S1 protein of reference strain LX4.

For the ANPEP–LX4 S1 complex, pose28 was identified as the optimal docking conformation, with a PIPER pose score of −26.603 and a PIPER pose energy of −1193.204 kcal/mol. Structural visualization showed stable complex formation between ANPEP (blue) and LX4 S1 (purple; Fig. 7). Interaction analysis demonstrated that ASN872 of ANPEP formed a hydrogen bond with MET1 of LX4 S1, while GLU578 formed both a hydrogen bond and a salt bridge with LYS571. In addition, TYR518 formed a hydrogen bond with LEU578, along with multiple additional stabilizing interactions. However, unlike the F210 strain, no detectable interactions were observed between residues 88–89 of the LX4 S1 protein and the ANPEP receptor.

Comparative analysis of the two docking complexes revealed a distinct difference in receptor-binding patterns. In the F210 isolate, residues 88–89 were directly involved in the interaction interface with ANPEP, whereas the corresponding region in the classical LX4 reference strain did not participate in receptor binding. These findings suggest that amino acid variations within the 88–89 region may alter the binding mode of the S1 protein to ANPEP, potentially affecting receptor recognition specificity and viral adaptation. Collectively, these results highlight the structural and functional importance of the 88–89 region in the S1 protein and provide mechanistic insight into the receptor-binding differences between the newly isolated strain F210 and the classical LX4 reference strain.

## Discussion

IB remains a critical limiting factor for the global poultry industry. The persistent epidemic status of IBV is primarily attributed to its high genetic variability, frequent homologous recombination, and robust capacity to evade host immune responses (Xu et al., 2019; Zhou et al., 2017). In recent years, commercial poultry breeds in Yunnan Province have been predominantly introduced from other Chinese provinces. Under the unique free-range breeding mode prevalent in Yunnan, externally introduced chickens may carry latent IBV strains. Close contact and mixed rearing between introduced commercial flocks and local indigenous chickens, combined with the heterogeneous immune backgrounds of indigenous poultry populations, substantially facilitates the emergence of novel IBV mutations and recombinant variants.

Viral positivity detection rates serve as core epidemiological indicators for evaluating IBV circulation intensity and disease burden in poultry populations. Although the overall positivity rate observed in this study was relatively low (0.89%), IBV was detected in both live poultry markets and breeding farms, indicating persistent circulation of the virus in Yunnan Province. These findings suggest that IBV distribution in Yunnan is heterogeneous and may be influenced by local rearing modes, poultry trading activities, and ecological factors. Widespread free-range farming of indigenous chickens in Yunnan enables frequent ecological contact between local poultry and commercial flocks circulated via live poultry markets, which greatly increases the opportunities for viral transmission and long-term viral maintenance in regional poultry populations. Notably, successful isolation of multiple genetically distinct IBV genotypes even under low overall prevalence strongly indicates continuous viral evolution and adaptive variation within local poultry flocks. Therefore, continued molecular surveillance is required to accurately evaluate the epidemiological status of IBV and identify emerging variants with potential implications for disease control.

Southwestern China has witnessed the co-circulation of multiple IBV genotypes in recent years, forming a complex and dynamic epidemiological landscape. Consistent with national epidemic trends, the GI-19 (QX) genotype is the predominant endemic strain in southwestern China with high detection frequency in southern provincial regions (Yan et al., 2021). Previous regional surveillance studies have also verified GI-19 as the dominant IBV lineage in southwestern China, accompanied by the co-circulation of multiple minor. The GVI-1 genotype (TC07-2 lineage) was first detected in southern China and subsequently spread to neighboring provinces, probably in association with interregional poultry movement; its molecular characteristics in China have been extensively investigated (Bo et al., 2022; Mase M et al., 2010; Ren et al., 2019). The close phylogenetic relationship between the Yunnan and Sichuan GI-19 strains, together with the 93.33%–96.91% nucleotide identity among Chinese GI-19 reference strains, supports regional circulation accompanied by genetic variation. The high sequence homology of several Yunnan IBV isolates with Guangxi and Henan strains further indicates potential cross-regional viral dissemination mediated by poultry trading activities. In particular, the high genetic similarity between the Yunnan GVI-1 isolate YX3 and Guangxi/Guangdong strains suggests a probable south-to-north viral introduction route into Yunnan, which aligns with the fact that most commercial broilers farmed in Yunnan are sourced from southern Chinese provinces. Mixed rearing of immunologically distinct indigenous and commercial chickens creates favorable conditions for viral co-infection, thereby promoting IBV genetic recombination. Additionally, as a key overwintering station along major migratory bird flyways, Yunnan Province faces persistent risks of cross-species avian viral transmission mediated by migratory wild birds (He et al., 2020; Li et al., 2019). Collectively, the endemic prevalence of IBV in Yunnan is shaped by dual mechanisms: long-term local viral circulation and frequent exogenous viral introduction from neighboring regions.

At the molecular level, this study identified a unique valine–glycine (V–G) amino acid insertion at residues 88–89 of the S1 protein in the field isolate F210. The hypervariable regions of the IBV S1 subunit are functionally critical, as they govern viral attachment to host cells, antigenic variation, specific receptor recognition, and viral tissue tropism (Fan et al., 2019; Parsons et al., 2019; Promkuntod et al., 2014). Accordingly, the unique 88–89 insertion detected in F210 may induce structural alterations and functional changes in the S1 protein. Protein–protein docking simulations revealed that the 88–89 residue region of F210 S1 directly participates in molecular interactions with the chicken host receptor ANPEP. In contrast, the corresponding region of the classical IBV LX4 strain exhibits no binding activity toward ANPEP in simulated complexes. Structural variations in the S1 subunit have been proven to alter viral receptor recognition and binding affinity (Li et al., 2016). Thus, the novel amino acid insertion in F210 may trigger localized conformational changes in the S1 receptor-binding domain, further modifying virus–receptor interaction patterns. Notably, molecular docking provides computational predictions rather than experimental evidence and cannot demonstrate that the insertion alters receptor binding, host adaptation, infectivity, pathogenicity, or host range. The modeled differences instead identify residues 88–89 as a candidate site for further investigation of IBV–host interactions. Future studies will include receptor-binding assays, reverse-genetics experiments, and infectivity and pathogenicity analyses to determine the biological significance of this insertion.

Recombination analysis revealed distinct recombination breakpoints within the *1a* region of isolates Q47,F13,F210 and YX3. Isolate YX3 additionally harbored recombination breakpoints in the *1b* regions. The 1*a* and 1b genomic regions are closely associated with viral host binding capability, genome replication efficiency, and antigenic characteristics (Quinteros et al., 2016; Yan et al., 2021;). And such recombination may influence viral genetic diversity, antigenicity, pathogenicity, and vaccine protection (Jiang et al., 2018). Multiple natural patterns among different IBV genotypes have been previously reported, including recombination between GI-13/GI-19 and GI-19/GI-15 lineages, as well as complex recombinant events involving GI-7, GI-13, and GI-19 strains(Chen et al., 2024; Kim et al., 2023; Huang et al., 2020). In this study, the recombination pattern involving the GI-22 strains A2-EU526388 and SAIBK-DQ288927 was detected in the 1*a* region of the GI-19 isolates Q47, F13, and F210 and also in the GVI-1 isolate YX3, suggesting that this region may be particularly active in recombination involving GI-22-related sequences. Additional recombination involving the 1*a* and 1*b* regions and GI-22- and GI-19-related parental sequences was identified in YX3 and may affect replication-associated functions or host adaptation (Jiang et al., 2018). GI-22-derived regions may also contribute replication-related advantages to recombinant strains (Ren et al., 2019). The frequent identification of GI-22 as a putative major or minor parent may reflect its regional circulation and the increased probability of co-infection with other lineages (Marandino et al., 2022). Recombination in these genomic regions has been associated with IBV diversification and spread (Hou et al., 2020). The use of GI-22-related strains in vaccine development may provide additional opportunities for gene exchange with circulating strains (van Beurden et al., 2018), potentially contributing to antigenic variation and immune escape (Quinteros et al., 2016). Given the previous endemic prevalence of GI-22 strains in southern China (Zhao et al., 2019), the dominant involvement of GI-22 in Yunnan IBV recombination events is closely related to its regional epidemic distribution and the unique mixed breeding mode of Yunnan poultry industry.

In conclusion, the present study enriches the current database of IBV genetic diversity in Yunnan Province and provides a fundamental basis for further exploring the epidemiological characteristics and biological significance of emerging local IBV variants. Meanwhile, several limitations of this study should be acknowledged. First, the limited sample size and incomplete regional sampling coverage may restrict the generalizability of the results to the entire province. Second, this study mainly focused on the genetic variation of the S1 gene, while comprehensive whole-genome analysis is required to fully elucidate the intricate recombination patterns and evolutionary dynamics of endemic IBV strains. Third, all functional analyses in this study rely on computational simulation, and the lack of in vitro and in vivo functional verification experiments leaves the effects of S1 amino acid mutations/insertions on viral antigenicity and pathogenicity unclarified.

Future research directions will focus on addressing the above deficiencies: expanding sampling scale and geographic coverage to systematically investigate the provincial prevalence of IBV; conducting comprehensive whole-genome sequencing to trace viral evolutionary and recombination patterns; performing targeted functional experiments to verify the biological roles of key variant loci; developing genotype-targeted vaccines for prevalent local IBV strains; and exploring the regulatory effects of indigenous chicken genetic characteristics on IBV evolution, mutation, and transmission. These efforts will facilitate the establishment of precise and targeted IBV prevention and control systems in Yunnan Province.

## Conclusion

In summary, this study isolated six IBV strains (five GI-19 and one GVI-1) from chickens in Yunnan Province. A unique valine–glycine insertion at positions 88–89 was identified in the S1 protein of isolate F210. Recombination analyses revealed frequent involvement of GI-22 lineage strains as parental donors, including cross-genotypic recombination among GI-22, GI-19, and GVI-1. Phylogenetic analysis showed close genetic relationships between Yunnan isolates and strains from Sichuan, Guangxi, and Henan. Notably, structural analysis suggested that variations in the S1 protein, particularly within the 88–89 region, may influence interactions with the host receptor ANPEP, highlighting a potential functional implication of these mutations. These findings provide baseline data on IBV genetic characteristics in Yunnan and underscore the importance of continuous molecular surveillance combined with functional analysis to better understand viral evolution and host adaptation.

## Authors’ contributions

**Weiwu Mu** designed the study, carried out the experiments, analyzed the data, prepared the figures, and wrote the initial draft of the manuscript. **Ronghai Wang** carried out the experiments, confirmed the results, and compiled and organized the data. **Xuzhao Zou** performed software-based analyses, processed the data, and compiled and organized the data. **Huixin Liu** assisted with part of the experimental work. **Jingxia Wang** prepared the figures and compiled and organized the data. **Xianghua Li** provided technical support for the methods. **Fengping He** performed software-based analyses and conducted data analysis. **Sunpeth Angkititrakul** revised and refined the manuscript. **Bin Xiang** revised and refined the manuscript and provided research resources. **Liangyu Yang**, as the corresponding author, designed and supervised the study, coordinated the research project, obtained financial support, and revised and refined the manuscript.All authors have reviewed and approved the final submitted manuscript.

## Funding

This study was funded by the Yunnan Province International Science and Technology Commissioner Recognition Work Project (202403AK140085), the Young Talent Project of the "Xing Dian Talent Support Program" of Yunnan Province (XDYC-QNRC-2023-0424), the Expert Workstation of Zhaotong (2025ZTYX016), and the Yunnan International Joint R&D Center of Intelligent Diagnosis and Prevention and Control of Transboundary Animal Diseases (202503AP140043).

## Disclosures

I wish to submit a Full-Length Article for publication in *Poultry Science,* titled “Molecular Characteristics, Phylodynamics, and Evolutionary Changes of Avian Infectious Bronchitis Virus detected from Chickens in Yunnan Province, 2021-2024.” The paper was coauthored by Weiwu Mu, Huixin Liu, Xuzhao Zou, Ronghai Wang, Jingxia Wang, Xianghua Li, Fengping He, Sunpeth Angkititrakul, Bin Xiang and Liangyu Yang.

The authors of the above-mentioned paper have no affiliations or associations with any organization/entity with any financial or non-financial interest in the subject matter or materials discussed in this manuscript.

## Footnotes

Supplementary material associated with this article can be found, in the online version, at doi:10.1016/j.psj.2026.107716.

## References

[bib0001] Bali K., Balint A., Farsang A., Marton S., Nagy B., Kaszab E., Belak S., Palya V., Banyai K. (2021). Recombination events shape the genomic evolution of infectious Bronchitis virus in Europe. Viruses.

[bib0002] Bo Z., Chen S., Zhang C., Guo M., Cao Y., Zhang X., Wu Y. (2022). Pathogenicity evaluation of GVI-1 lineage infectious bronchitis virus and its long-term effects on reproductive system development in SPF hens. Front. Microbiol..

[bib0003] Cavanagh D., Davis P.J., Cook J.K.A., Li D., Kant A., Koch G. (1992). Location of the amino acid differences in the S1 spike glycoprotein subunit of closely related serotypes of infectious bronchitis virus. Avian Pathol..

[bib0004] Chen H., Shi W., Feng S., Yuan L., Jin M., Liang S., Wang X., Si H., Li G., Ou C. (2024). A novel highly virulent nephropathogenic QX-like infectious bronchitis virus originating from recombination of GI-13 and GI-19 genotype strains in China. Poult. Sci..

[bib0005] Chen X., Mu W., Shao Y., Peng L., Zhang R., Luo S., He X., Zhang L., He F., Li L., Wang R., Yang L., Xiang B. (2024). Genetic and molecular characterization of H9N2 avian influenza viruses in Yunnan Province, Southwestern China. Poult. Sci..

[bib0006] Christian Drosten M.D. (2003). Identification of a novel coronavirus in patients with severe acute Respiratory syndrome. N. Engl. J. Med..

[bib0007] Cook J.K.A., Jackwood M., Jones R.C. (2012). The long view: 40 years of infectious bronchitis research. Avian Pathol..

[bib0008] de Wit J.J., Cook J.K.A., van der Heijden H.M.J.F. (2011). Infectious bronchitis virus variants: a review of the history, current situation and control measures. Avian Pathol..

[bib0009] Fan W., Chen J., Zhang Y., Deng Q., Wei L., Zhao C., Lv D., Lin L., Zhang B., Wei T., Huang T., Wei P., Mo M. (2022). Phylogenetic and spatiotemporal analyses of the complete genome sequences of Avian coronavirus infectious bronchitis virus in China during 1985–2020: revealing coexistence of multiple transmission chains and the origin of LX4-Type Virus. Front. Microbiol..

[bib0010] Fan W., Tang N., Dong Z., Chen J., Zhang W., Zhao C., He Y., Li M., Wu C., Wei T., Huang T., Mo M., Wei P. (2019). Genetic analysis of Avian coronavirus infectious bronchitis virus in yellow chickens in southern China over the past decade: revealing the changes of Genetic diversity, dominant genotypes, and selection pressure. Viruses.

[bib0011] Fellahi S., El Harrak M., Ducatez M., Loutfi C., Koraichi S.I., Kuhn J.H., Khayi S., El Houadfi M., Ennaji M.M. (2015). Phylogenetic analysis of avian infectious bronchitis virus S1 glycoprotein regions reveals emergence of a new genotype in Moroccan broiler chicken flocks. Virol. J..

[bib0012] He Y., Lu B., Dimitrov K.M., Liang J., Chen Z., Zhao W., Qin Y., Duan Q., Zhou Y., Liu L., Li B., Yu L., Duan Z., Liu Q. (2020). Complete genome sequencing, molecular epidemiological, and pathogenicity analysis of pigeon paramyxoviruses type 1 isolated in Guangxi, China during 2012-2018. Viruses.

[bib0013] Hou Y., Zhang L., Ren M., Han Z., Sun J., Zhao Y., Liu S. (2020). A highly pathogenic GI-19 lineage infectious bronchitis virus originated from multiple recombination events with broad tissue tropism. Virus. Res..

[bib0014] Huang M., Zou C., Liu Y., Han Z., Xue C., Cao Y. (2020). A novel low virulent respiratory infectious bronchitis virus originating from the recombination of QX, TW and 4/91 genotype strains in China. Vet. Microbiol..

[bib0015] Huo J.L., Wu G.S., Chen T., Huo H.L., Yuan F., Liu L.X., Ge C.R., Miao Y.W. (2014). Genetic diversity of local Yunnan chicken breeds and their relationships with Red Junglefowl. Genet. Mol. Res..

[bib0016] Ji J., Gao Y., Chen Q., Wu Q., Xu X., Kan Y., Yao L., Bi Y., Xie Q. (2020). Epidemiological investigation of avian infectious bronchitis and locally determined genotype diversity in central China: a 2016-2018 study. Poult. Sci..

[bib0017] Jiang L., Han Z., Chen Y., Zhao W., Sun J., Zhao Y., Liu S. (2018). Characterization of the complete genome, antigenicity, pathogenicity, tissue tropism, and shedding of a recombinant avian infectious bronchitis virus with a ck/CH/LJL/140901-like backbone and an S2 fragment from a 4/91-like virus. Virus. Res..

[bib0018] Jiang L., Zhao W., Han Z., Chen Y., Zhao Y., Sun J., Li H., Shao Y., Liu L., Liu S. (2017). Genome characterization, antigenicity and pathogenicity of a novel infectious bronchitis virus type isolated from south China. Infect. Genet. Evol..

[bib0019] Kim H.J., Lee H.C., Cho A.Y., Choi Y.J., Lee H., Lee D.H., Song C.S. (2023). Novel recombinant avian infectious bronchitis viruses from chickens in Korea, 2019-2021. Front. Vet. Sci..

[bib0020] Li F. (2016). Structure, function, and evolution of coronavirus spike proteins. Annu Rev. Virol..

[bib0021] Li X., Xu B., Shaman J. (2019). The impact of environmental transmission and epidemiological features on the geographical translocation of highly pathogenic Avian Influenza virus. Int. J. Env. Res. Public Health.

[bib0022] Li X., Zai J., Zhao Q., Nie Q., Li Y., Foley B.T., Chaillon A. (2020). Evolutionary history, potential intermediate animal host, and cross-species analyses of SARS-CoV-2. J. Med. Virol..

[bib0023] Liu S., Kong X. (2004). A new genotype of nephropathogenic infectious bronchitis virus circulating in vaccinated and non-vaccinated flocks in China. Avian Pathol..

[bib0024] Liu S., Xu Q., Han Z., Liu X., Li H., Guo H., Sun N., Shao Y., Kong X. (2014). Origin and characteristics of the recombinant novel avian infectious bronchitis coronavirus isolate ck/CH/LJL/111054. Infect. Genet. Evol..

[bib0025] Liu S., Zhang X., Wang Y., Li C., Han Z., Shao Y., Li H., Kong X. (2009). Molecular characterization and pathogenicity of infectious bronchitis coronaviruses: complicated evolution and epidemiology in China caused by cocirculation of multiple types of infectious bronchitis coronaviruses. Intervirology.

[bib0026] Ma T., Xu L., Ren M., Shen J., Han Z., Sun J., Zhao Y., Liu S. (2019). Novel genotype of infectious bronchitis virus isolated in China. Vet. Microbiol..

[bib0027] Marandino A., Vagnozzi A., Tomas G., Techera C., Gerez R., Hernandez M., Williman J., Realpe M., Greif G., Panzera Y., Perez R. (2022). Origin of new lineages by recombination and mutation in avian infectious bronchitis virus from South America. Viruses.

[bib0028] Martin D.P., Murrell B., Golden M., Khoosal A., Muhire B. (2015). RDP4: detection and analysis of recombination patterns in virus genomes. Virus. Evol..

[bib0029] Mase M., Nahoko K., Ootani Y. (2010). A novel genotype of avian infectious bronchitis virus isolated in Japan in 2009. J. Vet. Med. Sci..

[bib0030] Mendoza-Gonzalez L., Marandino A., Panzera Y., Tomas G., Williman J., Techera C., Gayosso-Vazquez A., Ramirez-Andoney V., Alonso-Morales R., Realpe-Quintero M., Perez R. (2022). Research note: high genetic diversity of infectious bronchitis virus from Mexico. Poult. Sci..

[bib0031] Molenaar R.J., Dijkman R., de Wit J.J. (2020). Characterization of infectious bronchitis virus D181, a new serotype (GII-2). Avian Pathol..

[bib0032] Miłek J., Blicharz-Domańska K. (2018). Coronaviruses in avian species - review with focus on epidemiology and diagnosis in wild birds. J. Vet. Res..

[bib0033] Parsons L.M., Bouwman K.M., Azurmendi H., de Vries R.P., Cipollo J.F., Verheije M.H. (2019). Glycosylation of the viral attachment protein of avian coronavirus is essential for host cell and receptor binding. J. Biol. Chem..

[bib0034] Promkuntod N., van Eijndhoven R.E., de Vrieze G., Grone A., Verheije M.H. (2014). Mapping of the receptor-binding domain and amino acids critical for attachment in the spike protein of avian coronavirus infectious bronchitis virus. Virology.

[bib0035] Quinteros J.A., Lee S.W., Markham P.F., Noormohammadi A.H., Hartley C.A., Legione A.R., Coppo M.J.C., Vaz P.K., Browning G.F. (2016). Full genome analysis of Australian infectious bronchitis viruses suggests frequent recombination events between vaccine strains and multiple phylogenetically distant avian coronaviruses of unknown origin. Vet. Microbiol..

[bib0036] Ren M., Sheng J., Ma T., Xu L., Han Z., Li H., Zhao Y., Sun J., Liu S. (2019). Molecular and biological characteristics of the infectious bronchitis virus TC07-2/GVI-1 lineage isolated in China. Infect. Genet. Evol..

[bib0037] van Beurden S.J., Berends A.J., Kramer-Kuhl A., Spekreijse D., Chenard G., Philipp H.C., Mundt E., Rottier P.J.M., Verheije M.H. (2018). Recombinant live attenuated avian coronavirus vaccines with deletions in the accessory genes 3ab and/or 5ab protect against infectious bronchitis in chickens. Vaccine.

[bib0038] Wang S., Pan J., Zhou K., Chu D., Li J., Chen Y., Qi Q., Wei S., Li C., Sui J., Wu F., Li J., Hou G., Liu H., Wang K. (2024). Characterization of the emerging recombinant infectious bronchitis virus in China. Front. Microbiol..

[bib0039] Xie Y., Fan W., Lin Z., Li J., Zhang T., Zhang Y., Liu L., Chen S., Dong X., Chen Q., Li H., Zhao W., Chen J., Lv D., Wang W., Mo M. (2025). Evolution and transmission dynamics of infectious bronchitis virus in southern China during 2018–2023: emergence of a novel GI-19 subtype and re-emergence of GI-28. Poult. Sci..

[bib0040] Xu L., Ren M., Sheng J., Ma T., Han Z., Zhao Y., Sun J., Liu S. (2019). Genetic and biological characteristics of four novel recombinant avian infectious bronchitis viruses isolated in China. Virus. Res..

[bib0041] Yan W., Qiu R., Wang F., Fu X., Li H., Cui P., Zhai Y., Li C., Zhang L., Gu K., Zuo L., Lei C., Wang H., Yang X. (2021). Genetic and pathogenic characterization of a novel recombinant avian infectious bronchitis virus derived from GI-1, GI-13, GI-28, and GI-19 strains in Southwestern China. Poult. Sci..

[bib0042] Zhang D., Gao F., Jakovlic I., Zou H., Zhang J., Li W.X., Wang G.T. (2020). PhyloSuite: an integrated and scalable desktop platform for streamlined molecular sequence data management and evolutionary phylogenetics studies. Mol. Ecol. Resour..

[bib0043] Zhao Y., Xie D., Zhang K., Cheng J., Xu G., Zhang G. (2019). Pathogenicity of a GI-22 genotype infectious bronchitis virus isolated in China and protection against it afforded by GI-19 vaccine. Virus. Res..

[bib0044] Zhou H., Zhang M., Tian X., Shao H., Qian K., Ye J., Qin A. (2017). Identification of a novel recombinant virulent avian infectious bronchitis virus. Vet. Microbiol..

