# Supplementary material for: Molecular characteristics, phylodynamics, and evolutionary changes of avian infectious bronchitis virus detected from chickens in Yunnan Province, 2021–2024
Source: Poult Sci. 2026 Sep 3;105(12):107716. doi: 10.1016/j.psj.2026.107716 (PMC13594865; doi:10.1016/j.psj.2026.107716)
Supplement: Supplementary file 1 [file mmc1.docx]

**Supplementary Table 1** **Primers used for genome amplification**

| Fragments | Location | Sense primer (5'–3') | Anti-sense primer (5'–3') | Annealed °C |
| --- | --- | --- | --- | --- |
| P1 | 75-1733 | GTTGCTAGTATCACTGCTTG | TTAAGTGCTGCCACAAGTTC | 55°C |
| P2 | 1544-3117 | GCTATGTCTTTAAGAGTGGT | CAAAAGTTTCTTCACCAGATGG | 53°C |
| P3 | 2689-4190 | GATACAGACCTCACAGTAGAACA | TCCTCAACATCATCTGCTGTA | 55°C |
| P4 | 4170-5486 | TACAGCAGATGATGTTGAGGA | TTAGGTTTATTAAGGTGTTCTG | 50°C |
| P5 | 5428-6772 | GATAAAATTGGTGGTGTAACTA | CTGCATTCTTTGCTTCCTC | 55°C |
| P6 | 5595-7445 | AGTAGGAGATGGTGTAGTTCG | GCAAATTCTCTACTCATTTCCC | 53°C |
| P7 | 7258-8962 | AGGTCTGGTGCTAAACAAGT | CCAACCACAATCCATTAAGA | 48°C |
| P8 | 8587-10463 | GGCAATGAGTTTGTTGGTAAT | GTAAACATCCAACCTATGCC | 51°C |
| P9 | 9346-10628 | CACCATCTTGAGTTACCTAAT | CCTTGTATAAGTATATTTGTA | 46°C |
| P10 | 10372-11856 | GCTAAGTGGATGCTGTATTA | AGCACCAAGTACCATACCTC | 53°C |
| P11 | 11615-13244 | GCAGCGTAGAGTCTAAATGT | ACAATCTCGACAATTAGGGTG | 53°C |
| P12 | 12872-14528 | AATGGGACCTATTGTTCGTCG | CTGCTGGTATAACTCATACTGC | 55°C |
| P13 | 14433-16243 | CCACATCTGCTAATGTTGC | ATGTCACAACTAACTTCTGGC | 53°C |
| P14 | 15989-17447 | CAGTGGTAAGTCACACTTTGC | GTCTGCTAACATCTGAACTATC | 51°C |
| P15 | 17203-18731 | TAGAAGCCACACATGCTTGTG | GTCTACACCTAAACCACTCAAT | 55°C |
| P16 | 18639-20094 | ACTCTGCCTACATCAGTTGC | ACATTGTCCACCATGCACAATC | 53°C |
| P17 | 19815-21419 | GTATCTGATGCACACGTGTCTGT | GAAACTGAAAGTGAATTAAACCA | 55°C |
| P18 | 21254-22834 | AAACAGCTCAGAGTGGTTATTA | CTTGAATTTGTTGTAATGCTAG | 50°C |
| P19 | 22752-24044 | AATTGCTGCTTCCTTTAATA | CTTCTAAGTTTAACATTAGTCTA | 50°C |
| P20 | 23955-25186 | CTAATACAAGTTTTACTCCAA | CTTCTAAGTTTAACATTAGTCTA | 48°C |
| P21 | 25070-26376 | GTGACCAAAGCGGAAATAAGA | TGATCTTCCACTCCTACCACG | 53°C |
| P22 | 26269-27608 | GATCCTGATAAGTTTGACCAATA | TGCTCTAACTCTATACTAGCCTATA | 52°C |
