# Supplementary material for: Molecular characteristics, phylodynamics, and evolutionary changes of avian infectious bronchitis virus detected from chickens in Yunnan Province, 2021–2024
Source: Poult Sci. 2026 Sep 3;105(12):107716. doi: 10.1016/j.psj.2026.107716 (PMC13594865; doi:10.1016/j.psj.2026.107716)
Supplement: Supplementary file 2 [file mmc2.docx]

**Supplementary Table2 HVR I Amino acid variation of the genes encoding surface proteins S1 of the GI-19 strans IBVs identified in this study**

| Isolate | Genotype | HVR I mutant amino acids | | | | | | | | | | | |  | S protein  cleavage  site |
| --- | --- | --- | --- | --- | --- | --- | --- | --- | --- | --- | --- | --- | --- | --- | --- |
|  |  | 35 | 38 | 39 | 44 | 53 | 54 | 55 | 56 | 58 | 62 | 65 | 68 | 88-89  Insertion |  |
| H120 | GI-1 | F | P | D | H | I | S | S | E | N | S | G | V | - | RRFRR |
| M41 | GI-1 | F | P | N | H | I | S | S | E | N | S | G | V | - | RRFRR |
| F210 | GI-19 | F | S | S | Q | V | S | S | R | N | A | E | V | VG | HRRRR |
| F13 | GI-19 | Y | P | N | H | V | T | N | Y | S | S | V | V | - | HRRRR |
| YJ1 | GI-19 | F | P | N | Q | S | T | N | Y | N | S | E | I | - | HRRRR |
| YJ2 | GI-19 | F | P | N | Q | S | T | N | Y | N | S | E | I | - | HRRRR |
| Q47 | GI-19 | Y | P | N | Q | V | T | N | Y | S | S | V | V | - | HRRRR |
