# Supplementary material for: Molecular characteristics, phylodynamics, and evolutionary changes of avian infectious bronchitis virus detected from chickens in Yunnan Province, 2021–2024
Source: Poult Sci. 2026 Sep 3;105(12):107716. doi: 10.1016/j.psj.2026.107716 (PMC13594865; doi:10.1016/j.psj.2026.107716)
Supplement: Supplementary file 3 [file mmc3.docx]

**Supplementary Table3** **HVR II and HVR III Amino acid variation of the genes encoding surface proteins S1 of the GI-19 strains IBVs identified in this study**

| Isolate | Genotype | HVR II mutant amino acids | | | | | | | | | | | HVR III mutant amino acids | | | | S protein  cleavage  site |
| --- | --- | --- | --- | --- | --- | --- | --- | --- | --- | --- | --- | --- | --- | --- | --- | --- | --- |
|  |  | 117 | 120 | 122 | 125 | 128 | 130 | 131 | 132 | 140 | 142 | 143 | 271 | 282 | 291 |  | |
| H120 | GI-1 | K | G | S | I | M | Q | Q | H | K | G | Q | T | G | Q | RRFRR | |
| M41 | GI-1 | K | G | S | I | M | Q | K | N | K | G | Q | T | G | Q | RRFRR | |
| F210 | GI-19 | A | S | K | L | L | P | Q | G | R | H | T | A | N | N | HRRRR | |
| F13 | GI-19 | S | T | S | I | M | A | R | D | K | G | S | V | N | N | HRRRR | |
| YJ1 | GI-19 | S | A | S | I | M | A | R | D | K | G | S | T | T | S | HRRRR | |
| YJ2 | GI-19 | S | A | S | I | M | A | R | D | K | G | S | T | T | S | HRRRR | |
| Q47 | GI-19 | S | T | S | I | M | A | R | D | K | G | S | V | N | Q | HRRRR | |
