# Supplementary material for: Molecular characteristics, phylodynamics, and evolutionary changes of avian infectious bronchitis virus detected from chickens in Yunnan Province, 2021–2024
Source: Poult Sci. 2026 Sep 3;105(12):107716. doi: 10.1016/j.psj.2026.107716 (PMC13594865; doi:10.1016/j.psj.2026.107716)
Supplement: Supplementary file 4 [file mmc4.docx]

**Supplementary Table 4** **HVR Amino acid variation of the genes encoding surface proteins S1 of the GVI-1 strains IBVs identified in this study**

| Isolate | Genotype | HVR I mutant amino acids | | | | | HVR II mutant amino acids | | | HVR III mutant amino acids | | | | | S protein  cleavage  site |
| --- | --- | --- | --- | --- | --- | --- | --- | --- | --- | --- | --- | --- | --- | --- | --- |
|  |  | 39 | 52 | 58 | 63 | 64 | 96 | 130 | 133 | 304 | 319 | 329 | 330 | 355 |  |
| YX3 | GVI-1 | T | R | N | G | D | T | K | A | T | S | T | K | V | HRRKR |
| CK/CH/GX/L1501-2 | GVI-1 | S | Q | S | S | E | N | R | D | I | R | M | R | 1 | HRHKR |
| CK/CH/HuB/HC1402-1 | GVI-1 | S | Q | S | S | E | N | R | D | I | R | M | R | 1 | HRHKR |
| GX-QZ130064 | GVI-1 | S | Q | S | S | E | N | R | E | I | R | M | S | 1 | RKRKR |
| GX-QZ130065 | GVI-1 | S | Q | S | S | E | N | R | E | I | R | M | S | 1 | HRRKR |
