# Supplementary material for: Molecular characteristics, phylodynamics, and evolutionary changes of avian infectious bronchitis virus detected from chickens in Yunnan Province, 2021–2024
Source: Poult Sci. 2026 Sep 3;105(12):107716. doi: 10.1016/j.psj.2026.107716 (PMC13594865; doi:10.1016/j.psj.2026.107716)
Supplement: Supplementary file 5 [file mmc5.docx]

**Supplementary Table 5 Recombination events of isolates as assessed using 7 different methods within the RPD4 software.**

|  | **Breakpoint positions** | | **Major parent** | | **Minor parent** | | ***Detection methods*** | | | | | | |
| --- | --- | --- | --- | --- | --- | --- | --- | --- | --- | --- | --- | --- | --- |
| **Strain** | **Begin** | **END** | **Strain** | **Similarity** | **Strain** | **Similarity** | R | G | B | M | C | S | T |
| F13 | 941 | 5681 | EU526388 | 89.3% | DQ288927 | 93.5% | **+** | **+** | **－** | **+** | **+** | **+** | **+** |
| F210 | 901 | 5701 | EU526388 | 80.85% | DQ288927 | 93% | **+** | **+** | **－** | **+** | **+** | **+** | **+** |
| Q47 | 941 | 5681 | EU526388 | 89.3% | DQ288927 | 93.4% | **+** | **+** | **－** | **+** | **+** | **+** | **+** |
| YX3 | 961 | 5681 | EU526388 | 75.6% | DQ288927 | 90.5% | **+** | **+** | **－** | **+** | **+** | **+** | **+** |
| YX3 | 5001 | 19841 | JF893452 | 86.4% | F13 | 98.5% | **+** | **+** | **+** | **+** | **+** | **+** | **+** |
